# Comparison of Automated Atlas-Based Segmentation Software for Postoperative Prostate Cancer Radiotherapy

**DOI:** 10.3389/fonc.2016.00178

**Published:** 2016-08-03

**Authors:** Grégory Delpon, Alexandre Escande, Timothée Ruef, Julien Darréon, Jimmy Fontaine, Caroline Noblet, Stéphane Supiot, Thomas Lacornerie, David Pasquier

**Affiliations:** ^1^Medical Physics Department, Institut de Cancérologie de l’Ouest, Centre René Gauducheau, Saint Herblain, France; ^2^Radiation Oncology Department, Centre Oscar Lambret, Lille, France; ^3^Medical Physics Department, SELARL d’imagerie médicale et de cancérologie du Pont Saint Vaast, Douai, France; ^4^Medical Physics Department, Institut Paoli-Calmettes, Marseille, France; ^5^Radiotherapy Department, Institut de Cancérologie de l’Ouest, Centre René Gauducheau, Saint Herblain, France; ^6^Medical Physics Department, Centre Oscar Lambret, Lille, France; ^7^CRISTAL UMR CNRS 9189, Lille University, Lille, France

**Keywords:** postoperative radiotherapy, prostate bed, atlas, automatic segmentation, contour comparison

## Abstract

Automated atlas-based segmentation (ABS) algorithms present the potential to reduce the variability in volume delineation. Several vendors offer software that are mainly used for cranial, head and neck, and prostate cases. The present study will compare the contours produced by a radiation oncologist to the contours computed by different automated ABS algorithms for prostate bed cases, including femoral heads, bladder, and rectum. Contour comparison was evaluated by different metrics such as volume ratio, Dice coefficient, and Hausdorff distance. Results depended on the volume of interest showed some discrepancies between the different software. Automatic contours could be a good starting point for the delineation of organs since efficient editing tools are provided by different vendors. It should become an important help in the next few years for organ at risk delineation.

## Introduction

Prostate bed radiotherapy after radical prostatectomy may present some clinical benefits in term of clinical outcome (1, 2). Although intraoperative irradiation is a possible treatment modality (3), irradiation is mainly delivered by external beam radiotherapy. Advances in radiation oncology led to intensity-modulated radiotherapy (IMRT) and image-guided radiotherapy (IGRT). Those advances allow to either increase dose to target tissues or spare surrounding healthy structures. The development of state-of-the-art technologies including imaging modalities, treatment planning systems, and linacs have enabled radiotherapy treatments to be highly specific (4). In this context, the delineation of target and normal organs is the prerequisite inputs to the planning process. Consequently, the implementation of modern radiotherapy treatment plans focuses on the need of contouring guidelines (5). A recent development in radiotherapy is the use of automated atlas-based auto-segmentation algorithms to aid in organ delineation (6). The aim of the study was to compare the different atlas-based auto-segmentation software available when used for prostate bed and organs at risk. The study was limited to a single radiation oncologist to avoid inter-rater variations. Indeed, significant levels of interobserver variability in target volume delineation have been demonstrated in prostate cancer radiotherapy (7–10). This variability is the most important source of uncertainties in radiotherapy (11, 12). However, this variability is out of the scope of our study as at least four consensuses originating from four scientific groups were validated (13). Therefore, no ground truth can be considered. The aim of our study was to assess how segmentation software are able to learn from the single radiation oncologist habits in order to reproduce these habits to novel patients.

## Materials and Methods

### Population and Treatment

Twenty consecutive patients, treated in a clinical center, were included in this study from January to September 2015 for a pT3aR0-R1N0M0 prostate cancer after surgery. They were treated by postoperative salvage IMRT. Treatment aimed at delivering 66 Gy to the prostatic bed as clinical target volume (CTV) (1). Computed Tomography scans (CT) were contoured by only one physician according to the Radiation Therapy Oncology Group (RTOG) guidelines for target volumes (5). The following organs at risk were also delineated: bladder, rectum, and femoral heads (14).

### Ethics

As French laws (data, data-collection, and freedom law, January, 6, 1978) agreed for single-center retrospective study, no specific written informed consent is needed. All patients have been orally informed about potential use of already recorded data for potential study.

### Atlas-Based Auto-Segmentation Software

Five software were compared. WorkFlow Box (Mirada Medical) (WFB), MIM Maestro (MIM Software), SPICE (Philips), ABAS (Elekta), and the atlas-based segmentation module included in RayStation (RaySearch Laboratories). WFB is a black-box server that performs atlas-based contouring automatically. WFB fits seamlessly in to your current process *via* standard DICOM protocols. WFB uses deformable registration algorithm to automatically apply contours to planning CTs based on multiple expert atlases.

Alternatively, clinicians can define their own atlases. In the current study, atlases were based on patient contours delineated by the expert physician. Auto-contouring is a feature of MIM Maestro software. Automatic contours may be based on either user-defined atlas libraries or automatic atlas subject selection. This software includes features to sort atlases depending on TNM status, lesion laterality, or physician. If several atlases are chosen to start the auto-segmentation, a structure set was generated per atlas, and data were gathered to create the simultaneous truth and performance level estimation (STAPLE) contours for each organ. STAPLE is an expected maximization algorithm that computes a probabilistic estimate of the true segmentation by weighting each segmentation on its estimated performance level (15). In addition, it provides tools to correct auto-contours and a scripting platform. ABAS (Elekta) approximates the anatomy contours by scanning a library of reference images, applying elements of those forms to a new patient image, and creating a structure set to fit the patient’s anatomy. The user may either choose an atlas among the library or use the STAPLE algorithm. In this study, the STAPLE algorithm was used. The operator cannot see or edit the contours within ABAS, but contours may be imported in any contouring solution, such as Focal or Monaco considering Elekta software. SPICE (Philips) that stands for Smart Probabilistic Image Contouring Engine, is an option of Pinnacle, a treatment planning system. This system computes contours from a probabilistic segmentation based on its own expert atlases, and the user cannot import his datasets to create another expert library. Consequently, only a limited number of treatment sites and organs is available. The transformation is based on a dense deformable registration method (Enhanced Demons), which further initializes organ-specific deformable models. The method is based on adaptation and probabilistic refinement (16). In addition to plan design and optimization features, RayStation Treatment Planning System (RS) provides an auto-segmentation solution based on ANAtomically CONstrained Deformation Algorithm (ANACONDA). ANACONDA combines image information (i.e., intensities) with anatomical information as provided by contoured image sets (17). It is a hybrid algorithm due to the combination of using image similarity and anatomical information. Model-based segmentation (MBS) and atlas-based segmentation (ABS) are available. MBS includes models with adjustable shape, size, and property parameters provided by RayStation for the different organs at risk, including femoral heads and bladder. ABS requires user-defined atlases with image sets and contours. In this study, only ABS was used, even for femoral heads and bladder.

### Atlas and Evaluation Databases

The first 10 patients were selected to build the atlas database except for SPICE that is working differently and used its own atlas database. The 10 following patients constituted the evaluation database. The aim of the study was to compare the contours produced by the different automatic tools against the physician contours. For each patient of the evaluation database, atlas-based auto-segmentation software produced a DICOM Structure Set using the provided atlas database. Automatic contours without any modification were then exported in DICOM format for the comparison.

### Contour Comparison

CTV, bladder, rectum, and femoral heads delineated by the physician and computed by the automatic tools were imported in DICOM format in the Slicer open source freeware (http://www.slicer.org). Automatic and expert contours defined on the different CT slices constituted volumes. The additional module DICOM RT was used to compare those volumes. Physician contours were used as reference contours. Different metrics were calculated to quantify the similarity between the automatic and the expert volumes.

The simple ratio *R* of the automatic volume (in cubic centimeter) divided by the expert volume (in cubic centimeter) was calculated.

The Dice Similarity Coefficient (DSC) was used to quantify the overlap between the expert and the automatic contours (18). DSC corresponds to the ratio of two times the intersection of two volumes divided by the sum of the two volumes (Eq 1).

(1) $$\text{DSC}=\frac{2\times \left|A\cap B\right|}{\left|A\right|+\left|B\right|}$$

where, *A* and *B* are the two volumes to be compared.

The Hausdorff distance (95% confidence interval) was used to quantify the magnitude of gross deviations between contour surfaces (19). The Hausdorff distance computation utilizes a maximum–minimum function as defined by Eq 2:

(2) $$h(a,b)={\text{max}}_{a\in A}\left\{{\text{min}}_{b\in B}\left\{d(a,b)\right\}\right\}$$

where *a* and *b* are points of contour sets *A* and *B*, and *d*(*a*,*b*) is the Euclidian distance between *a* and *b*. The Hausdorff distance (95% confidence interval) is calculated from the set H, which is composed of calculated Hausdorff distance *h*(*a*,*b*) values for all contour vertices of a contour set A. The value recorded *H*_95%_ is the largest distance that falls within the 95% confidence interval for the set of distances in *H*. The use of *H*_95%_ value minimizes the impact of large outliers in the Hausdorff distance calculation on the overall data (19).

## Results

For the 10 patients included in the evaluation dataset, the results are presented volume of interest by volume of interest.

**Figure 1:**
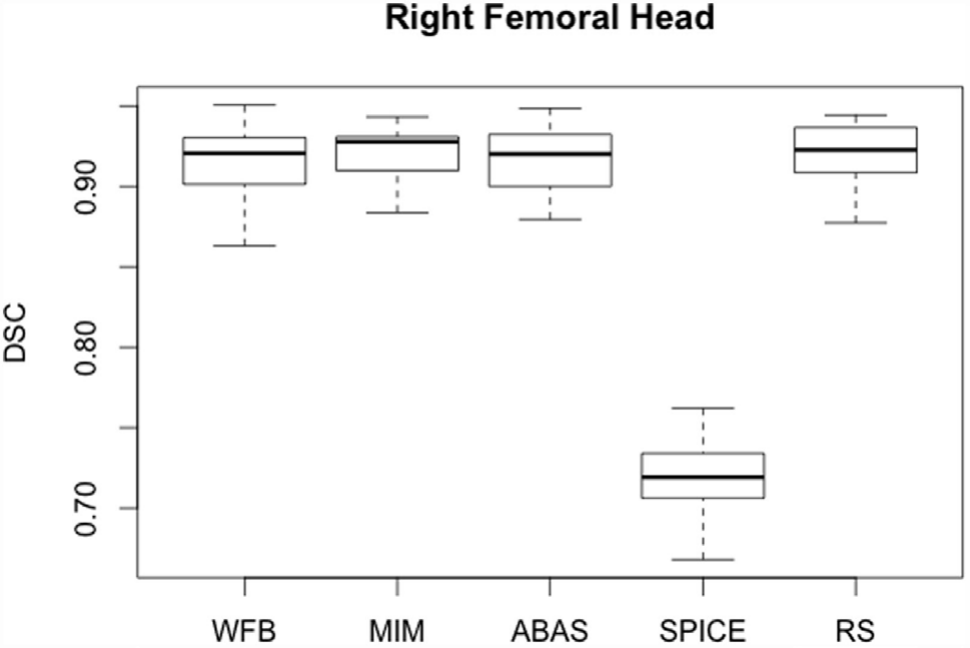
**Boxplots obtained for the dice similarity coefficient analysis of the right femoral head between the reference contours defined by the physician and the automatic contours computed by the different softwares**.

For femoral heads, results were obviously similar for the left and the right sides (Table 1). *R* values were higher than 0.93, except for SPICE. But for this latter, the problem was that femoral heads were automatically delineated on too many slices. The lowest slice on which a SPICE contour was defined differed from the expert. Those results were confirmed by the DSC analysis. Results were really consistent from one patient to another (Figure 1). Except for SPICE, DSC and *H*_95%_ were, respectively, about 0.90 and less than 10 mm for both femoral heads with small discrepancies whatever the patient. Femoral heads contours were acceptable, and only slight corrections would have been necessary to validate the automatic segmentation.

**Table 1:** **Results obtained for the evaluation dataset with the five commercial solutions [WFB (Mirada Medical), MIM (MIM Software), SPICE (Philips), ABAS (Elekta), and RS (RayStation)] compared to expert delineation for both femoral heads**.

		WFB	MIM	ABAS	SPICE	RS
Left femoral head	*R*	0.93 ± 0.06	0.96 ± 0.13	0.96 ± 0.06	0.59 ± 0.06	0.98 ± 0.09
	DSC mean	0.89 ± 0.05	0.89 ± 0.08	0.91 ± 0.04	0.70 ± 0.05	0.91 ± 0.03
	DSC median	0.91	0.91	0.92	0.72	0.92
	*H*_95%_ (mm)	9.2 ± 6.4	9.9 ± 7.9	8.6 ± 6.9	29.7 ± 9.0	8.8 ± 7.2
Right femoral head	*R*	0.93 ± 0.05	0.97 + 0.07	0.95 ± 0.05	0.60 ± 0.04	1.01 ± 0.07
	DSC mean	0.91 ± 0.03	0.92 ± 0.02	0.92 ± 0.02	0.72 ± 0.03	0.92 ± 0.02
	DSC median	0.92	0.93	0.92	0.72	0.92
	*H*_95%_ (mm)	8.1 ± 5.6	8.2 ± 5.3	8.5 ± 6.1	30.0 ± 6.5	6.4 ± 5.0

*R is the volume ration, DSC is the Dice Similarity Coefficient, and H_95%_ is the Hausdorff distance*.

**Figure 2:**
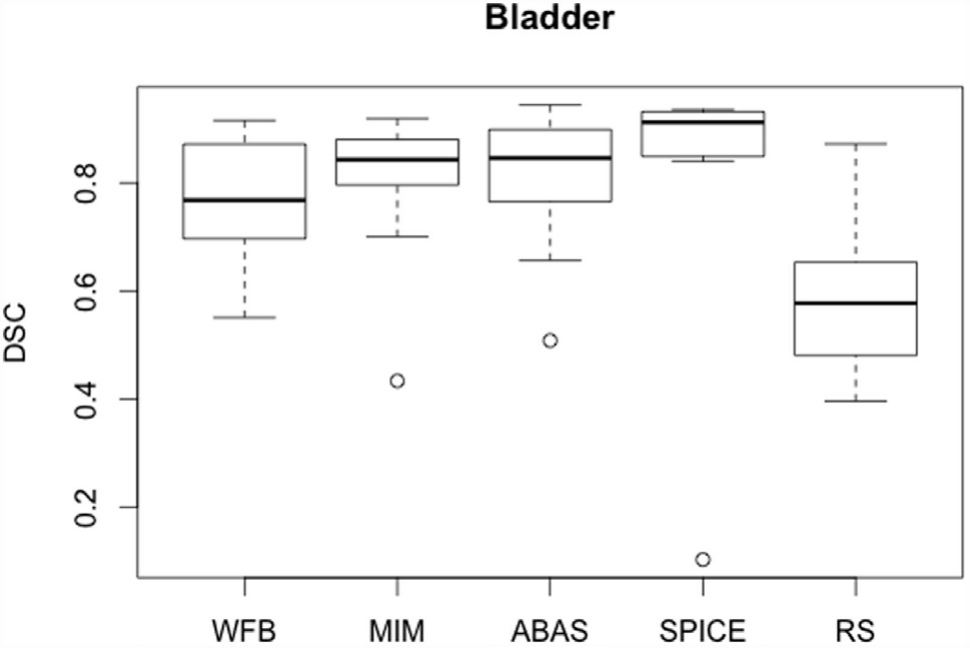
**Boxplots obtained for the DSC analysis of the bladder between the reference contours defined by the physician and the automatic contours computed by the different sotwares**.

Bladder *R* values were larger than those obtained for femoral heads, and differences were observed between patients and software (Table 2). SD was very large whatever the automatic solution. However, lower values were obtained with WFB and SPICE. Probably results would have been improved if CT scans had been injected with some contrast product. But DSC were satisfactory for most algorithms, with an average value higher than 0.75. For most algorithms, results were degraded by one or two cases. For example, SPICE median DSC was higher than 0.90, but average value was only 0.76 due to a very bad contour for Patient 10 (Figure 2). Similarly, ABAS and MIM failed for Patients 2 and 3. *H*_95%_ was about 15 mm, except for RS. RaySearch results were disappointing, but the MBS option was not used for this study. Automatic contours were globally satisfactory for most algorithms. However, results really depended on the patient case. Verification and corrections were required.

**Table 2:** **Results obtained for the evaluation dataset with the five commercial solutions [WFB (Mirada Medical), MIM (MIM Software), SPICE (Philips), ABAS (Elekta), and RS (RayStation)] compared to expert delineation for the bladder**.

	WFB	MIM	ABAS	SPICE	RS
*R*	1.01 ± 0.42	1.49 ± 0.77	1.31 ± 0.48	0.89 ± 0.31	1.62 ± 0.69
DSC mean	0.76 ± 0.12	0.80 ± 0.14	0.81 ± 0.13	0.76 ± 0.26	0.59 ± 0.15
DSC median	0.77	0.84	0.85	0.91	0.58
*H*_95%_ (mm)	15.0 ± 9.0	14.0 ± 6.3	13.6 ± 7.9	9.2 ± 11.7	28.5 ± 13.1

*R is the volume ration, DSC is the Dice Similarity Coefficient, and H_95%_ is the Hausdorff distance*.

**Figure 3:**
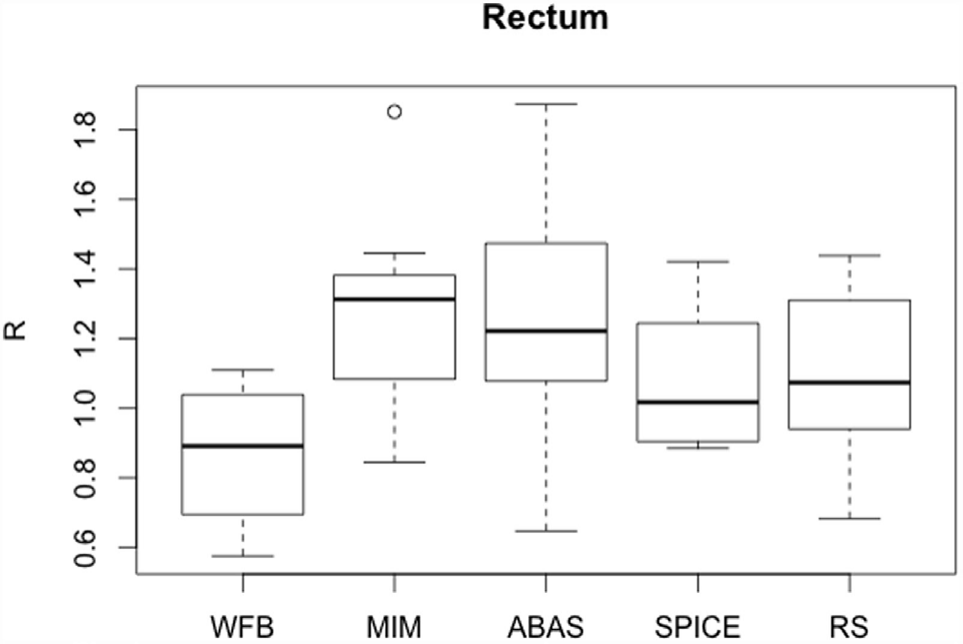
**Boxplots obtained for the *R* analysis of the rectum between the reference volumes defined by the physician and the automatic volumes computed by the different sotwares**.

**Figure 4:**
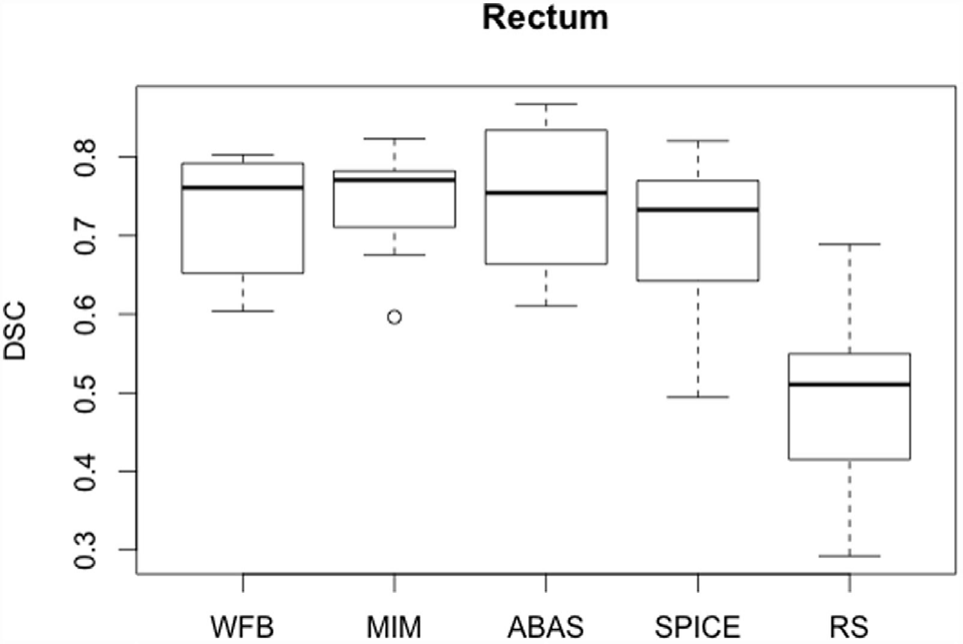
**Boxplots obtained for the DSC analysis of the rectum between the reference contours defined by the physician and the automatic contours computed by the different sotwares**.

Rectum *R* values were lower than those obtained for bladder, but SDs were still high, about 30% (Table 3). Rectum automatic contours were larger than expert contours, except for WFB (Figure 3). Despite the lower *R* values, DSC mean values were slightly lower than for bladder. However, less discrepancies were observed between patients, average, and median DSC were approximately equal. Globally, DSC results were similar for the different algorithms, except RS (Figure 4). *H*_95%_ was in the same order of magnitude, less than 15 mm, except for RS. Atlas-based contours presented discrepancies with the expert, and manual corrections were necessary.

**Table 3:** **Results obtained for the evaluation dataset with the five commercial solutions [WFB (Mirada Medical), MIM (MIM Software), SPICE (Philips), ABAS (Elekta), and RS (RayStation)] compared to expert delineation for the rectum**.

	WFB	MIM	ABAS	SPICE	RS
*R*	0.87 ± 0.19	1.27 ± 0.28	1.27 ± 0.38	1.30 ± 0.34	1.08 ± 0.28
DSC mean	0.73 ± 0.07	0.75 ± 0.07	0.75 ± 0.09	0.68 ± 0.12	0.49 ± 0.12
DSC median	0.76	0.77	0.75	0.73	0.51
*H*_95%_ (mm)	10.0 ± 3.0	9.9 ± 3.4	9.9 ± 4.4	13.0 ± 4.9	16.5 ± 3.7

*R is the volume ration, DSC is the dice similarity coefficient, and H_95%_ is the Hausdorff distance*.

**Figure 5:**
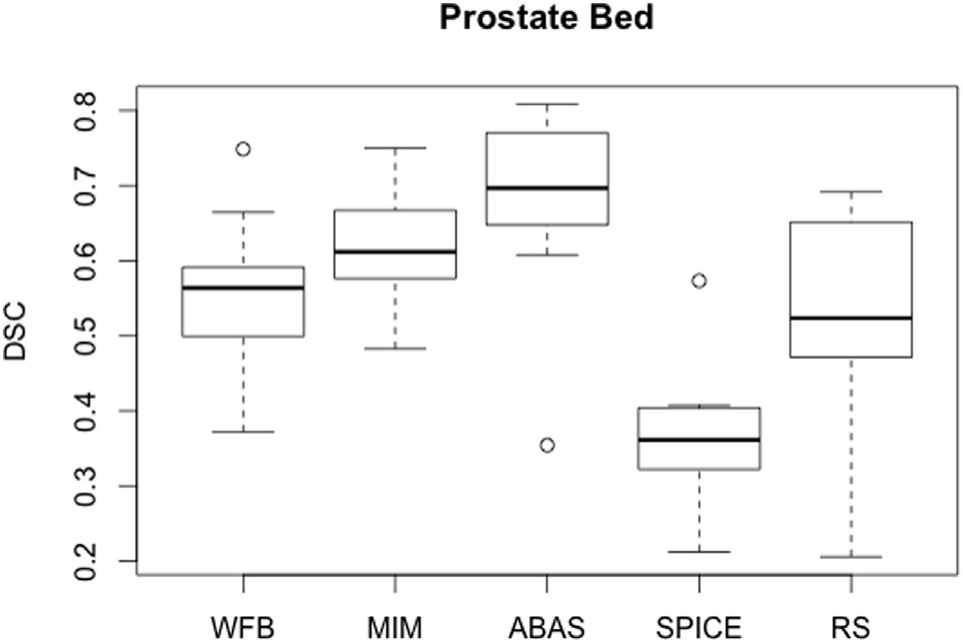
**Boxplots obtained for the DSC analysis of the prostate bed between the reference contours defined by the physician and the automatic contours computed by the different softwares**.

Automatic prostate bed contours were less satisfactory with large volume variations (Table 4). *R* values varied from 0.49 for SPICE to 1.37 for MIM. DSC was lower than 0.70 for all solutions, demonstrating that prostate bed cannot be automatically defined (Figure 5). Many corrections would be required to adapt automatic contours. However, ABAS had the best average DSC (Figure 5). Automatic prostate bed contours were insufficient. Manual segmentation should be preferred for this target volume whatever the algorithm.

**Table 4:** **Results obtained for the evaluation dataset with the five commercial solutions [WFB (Mirada Medical), MIM (MIM Software), SPICE (Philips), ABAS (Elekta), and RS (RayStation)] compared to expert delineation for the prostate bed CTV**.

	WFB	MIM	ABAS	SPICE	RS
*R*	0.53 ± 0.11	1.37 ± 0.35	1.04 ± 0.13	0.49 ± 0.15	0.82 ± 0.12
DSC mean	0.56 ± 0.10	0.61 ± 0.09	0.67 ± 0.13	0.37 ± 0.09	0.51 ± 0.17
DSC median	0.56	0.61	0.70	0.35	0.52
*H*_95%_ (mm)	11.9 ± 3.5	11.4 ± 4.0	8.4 ± 3.0	15.3 ± 2.6	12.4 ± 3.4

*R is the volume ration, DSC is the dice similarity coefficient, and H_95%_ is the Hausdorff distance*.

## Conclusion

To the best of our knowledge, no other study compared automatic delineation software for prostate cancer in the postoperative setting. The comparison of five different automatic-based segmentation software used for prostate bed and nearby organs showed these algorithms were very efficient for high contrast organs such as femoral heads. For other organs at risk, results were nuanced. Automatic contours were quite close to the expert contours, but corrections were required and for some cases, depending on the algorithm, computed contours were bad. Prostate bed contours were insufficient, but automatic segmentation aims essentially to delineate organs at risk. Postoperative CTV can be considered as a virtual volume without difference in terms of contrast or gray level over a large part of its volume. This difference compared to automatic prostate delineation may explain the bad outcomes in postoperative situation. A study shortcoming was the limited number of patients used to create the reference database. But the objective was mainly to compare the different software with the same settings, except for SPICE that considered its own reference datasets. In this context, a single physician defined the reference contours, and an arbitrary choice of 10 patients was done. For each automatic delineation software, an optimization study may lead to a different number of patients to build the reference database. Such studies may improve the coherence between automatic and physician contours (20). For example, RayStation recommends the use of up to 20 cases for atlas creation. However, results were consistent with the study published by Hwee et al. (6) that focused on MIM solution. Although proposed contours differed from one algorithm to another, the present study cannot establish a ranking of the software. Indeed, only 10 cases delineated by a single physician were selected to create the expert database, and 10 other cases were used for evaluation. In addition, this study did not consider the extra features proposed by some tools to modify the computed segmentation. Nevertheless, it allowed to state that atlas-based automatic segmentation has reached an interesting level of accuracy, especially for high contrast organs. Automatic contours could be a good starting point for the delineation of organs since efficient editing tools are provided by different vendors. It should become an important help in the next few years for organ at risk delineation.

## Author Contributions

AE, SS, and DP selected the patients and delineated the volumes of interest. GD, TR, JD, and TL generated the automatic contours. GD, JF, and CN analyzed the data. All authors contributed to the redaction of the manuscript.

## Conflict of Interest Statement

The authors declare that the research was conducted in the absence of any commercial or financial relationships that could be construed as a potential conflict of interest.

## References

[1] Bolla M Collette L pT3N0M0 prostate cancer: a plea for adjuvant radiation. Nat Rev Urol (2009) 6(8):410–2. 10.1038/nrurol.2009.103 19657374

[2] Thoms J Goda JS Zlotta AR Fleshner NE van der Kwast TH Supiot S Neoadjuvant radiotherapy for locally advanced and high-risk prostate cancer. Nat Rev Clin Oncol (2010) 8(2):107–13. 10.1038/nrclinonc.2010.207 21178999

[3] Buge F Chiavassa S Hervé C Rigaud J Delpon G Supiot S. Preclinical evaluation of intraoperative low-energy photon radiotherapy using spherical applicators in locally advanced prostate cancer. Front Oncol (2015) 5:204. 10.3389/fonc.2015.00204 26442216 PMC4569969

[4] Jaffray DA. Image-guided radiotherapy: from current concept to future perspectives. Nat Rev Clin Oncol (2012) 9(12):688–99. 10.1038/nrclinonc.2012.194 23165124

[5] Michalski JM Lawton CA El-Naqa I Ritter M O’Meara E Seider MJ Development of RTOG consensus guidelines for the definition of the clinical target volume for postoperative conformal radiation therapy for prostate cancer. Int J Radiat Oncol Biol Phys (2010) 76(2):361–8. 10.1016/j.ijrobp.2009.02.006 19394158 PMC2847420

[6] Hwee J Louie AV Gaede S Bauman G D’Souza D Sexton T Technology assessment of automated atlas based segmentation in prostate bed contouring. Radiat Oncol (2011) 6:110. 10.1186/1748-717X-6-110 21906279 PMC3180272

[7] Lawton CA Michalski JM El-Naqa I Kuban D Lee WR Rosenthal SA Variation in the definition of clinical target volumes for pelvic nodal conformal radiation therapy for prostate cancer. Int J Radiat Oncol Biol Phys (2009) 74(2):377–82. 10.1016/j.ijrobp.2008.08.003 18947941 PMC2905162

[8] Lawton CA Michalski JM El-Naqa I Buyyounouski MK Lee WR Menard C RTOG GU Radiation oncology specialists reach consensus on pelvic lymph node volumes for high-risk prostate cancer. Int J Radiat Oncol Biol Phys (2009) 74(2):383–7. 10.1016/j.ijrobp.2008.08.002 18947938 PMC2905150

[9] Livsey JE Wylie JP Swindell R Khoo VS Cowan RA Logue JP. Do differences in target volume definition in prostate cancer lead to clinically relevant differences in normal tissue toxicity? Int J Radiat Oncol Biol Phys (2004) 60(4):1076–81. 10.1016/j.ijrobp.2004.05.005 15519777

[10] Mitchell DM Perry L Smith S Elliott T Wylie JP Cowan RA Assessing the effect of a contouring protocol on postprostatectomy radiotherapy clinical target volumes and interphysician variation. Int J Radiat Oncol Biol Phys (2009) 75(4):990–3. 10.1016/j.ijrobp.2008.12.042 19345515

[11] Jameson MG Holloway LC Vial PJ Vinod SK Metcalfe PE. A review of methods of analysis in contouring studies for radiation oncology. J Med Imaging Radiat Oncol (2010) 54(5):401–10. 10.1111/j.1754-9485.2010.02192.x 20958937

[12] Ost P De Meerleer G Vercauteren T De Gersem W Veldeman L Vandecasteele K Delineation of the postprostatectomy prostate bed using computed tomography: interobserver variability following the EORTC delineation guidelines. Int J Radiat Oncol Biol Phys (2011) 81(3):e143–9. 10.1016/j.ijrobp.2010.12.057 21377287

[13] Malone S Croke J Roustan-Delatour N Belanger E Avruch L Malone C Postoperative radiotherapy for prostate cancer: a comparison of four consensus guidelines and dosimetric evaluation of 3D-CRT versus tomotherapy IMRT. Int J Radiat Oncol Biol Phys (2012) 84(3):725–32. 10.1016/j.ijrobp.2011.12.081 22444999

[14] Beckendorf V Bachaud J-M Bey P Bourdin S Carrie C Chapet O Target-volume and critical-organ delineation for conformal radiotherapy of prostate cancer: experience of French dose-escalation trials. Cancer Radiother (2002) 6(Suppl 1):78s–92s. 10.1016/S1278-3218(02)00217-2 12587386

[15] Warfield SK Zou KH Wells WM. Simultaneous truth and performance level estimation (STAPLE): an algorithm for the validation of image segmentation. IEEE Trans Med Imaging (2004) 23(7):903–21. 10.1109/TMI.2004.828354 15250643 PMC1283110

[16] Pekar V McNutt TR Kaus MR. Automated model-based organ delineation for radiotherapy planning in prostatic region. Int J Radiat Oncol Biol Phys (2004) 60:973–80. 10.1016/S0360-3016(04)00964-2 15465216

[17] Weistrand O Svensson S. The ANACONDA algorithm for deformable image registration in radiotherapy. Med Phys (2015) 42(1):40–53. 10.1118/1.4894702 25563246

[18] Dice LR Measures of the amount of ecologic association between species. Ecology (1945) 26:297–302. 10.2307/1932409

[19] Gardner SJ Wen N Kim J Liu C Pradhan D Aref I Contouring variability of human- and deformable-generated contours in radiotherapy for prostate cancer. Phys Med Biol (2015) 60(11):4429–47. 10.1088/0031-9155/60/11/4429 25988718

[20] Larrue A Gujral D Nutting C Gooding M The impact of the number of atlases on the performance of automatic multi-atlas contouring. Phys Med (2015) 31(Suppl 2):e30 10.1016/j.ejmp.2015.10.020

